# The Effect of Mulligan Mobilization Technique in Older Adults with Neck Pain: A Randomized Controlled, Double-Blind Study

**DOI:** 10.1155/2018/2856375

**Published:** 2018-05-15

**Authors:** Oznur Buyukturan, Buket Buyukturan, Senem Sas, Caner Karartı, İsmail Ceylan

**Affiliations:** ^1^School of Physical Therapy and Rehabilitation, Ahi Evran University, Kırşehir, Turkey; ^2^Department of Physical Medicine and Rehabilitation, Ahi Evran University Training and Research Hospital, Kırşehir, Turkey

## Abstract

**Background:**

The purpose of this study was to examine the effect of Mulligan mobilization technique (MMT) on pain, range of motion (ROM), functional level, kinesiophobia, depression, and quality of life (QoL) in older adults with neck pain (NP).

**Methods:**

Forty-two older adults with NP were included in the study, and they were randomly divided into two groups: traditional physiotherapy (TP) group and traditional physiotherapy-Mulligan mobilization (TPMM) group. Treatment program was scheduled for 10 sessions. Participants were assessed in terms of pain, ROM, functional level, kinesiophobia, depression, and QoL both pre- and posttreatment.

**Results:**

Pain, ROM, functional level, kinesiophobia, depression, and QoL improved in both groups following treatment (*p* < 0.05). When comparing effects of these two treatment programs, it was observed that the TPMM group had a better outcome (*p* < 0.05) in terms of ROM, kinesiophobia, depression, and QoL.

**Conclusion:**

In older adults with NP, MMT has been found to have significant effects on pain, ROM, functional level, kinesiophobia, depression, and QoL as long as it is performed by a specialist. “This trial is registered with NCT03507907”.

## 1. Introduction

Neck pain (NP) is one of the common musculoskeletal problems. NP can be caused by the stress over the musculoskeletal system due to postural disorders and may also be associated with other causes such as intervertebral disc herniation, nerve compression, or fracture [1]. The prevalence of NP is reported to range from 43% to 66.7%, which increases along with aging [2]. In a study conducted by March et al., on individuals over 65 years of age, the prevalence of NP was found to be 38.7% [3].

The use of various methods of manual treatments such as exercise, mobilization, and manipulation is supported by recent reviews on conservative treatments for mechanical NP [4]. Mulligan is one of the mobilization techniques that can be applied in case of NP. Being an important treatment tool used by most of the manual physical therapists, Mulligan mobilization techniques (MMTs) include several methods such as sustained natural epiphyseal glides (SNAGs) and natural epiphyseal glides that target the spine [5]. An immediate improvement in pain-free range of motion (ROM) in the involved joints is reposted as a result of applying this treatment approach [5, 6]. As a successful treatment approach for various orthopedics dysfunctions, a combination of the MMT concept along with several other methods of manual therapy has been suggested by the literature [7]. However, the application of the MMT for nonspecific NP in the older adults has not been investigated.

When the literature is examined, there is no randomized controlled study investigating the effect of the MMT on older adults with NP. This study aims to investigate the effect of Mulligan mobilization technique on pain, range of motion, functional level, kinesiophobia, fear of movement, depression, and quality of life in older adults with neck pain.

## 2. Material and Methods

### 2.1. Study Design

This study was designed as a randomized controlled, double-blinded study. Patients who agreed to participate in the study were divided into two groups—as the traditional physiotherapy (TP) group and traditional physiotherapy-Mulligan mobilization (TPMM) group—using a matched randomization method based on gender and age. Both the researchers performing the assessment (CK) and the treatment (OB) and the participants were blind about the groups. All assessments were made by the same investigator (CK) before and after treatment.

### 2.2. Participants

Individuals older than 65 years of age with NP, who were referred to the Physical Therapy and Rehabilitation Center of Ahi Evran University by a physiatrist (SS), were included in this study. Ongoing NP for at least 3 months having no neurological, rheumatological, or musculoskeletal problems and had not taken any analgesic medication for neck pain for the last 3 months were the inclusion criteria of the study. Exclusion criteria, however, were as follows: NP originating from various pathologies (tumor, rheumatoid arthritis, ankylosing spondylitis, fracture, dislocation, etc.), presence of cord compression, vertebrobasilar artery insufficiency, severe radiculopathy, osteoporosis or osteopenia (*t* score > −1), long-term use of anticoagulant or corticosteroid drugs, and patients who had received any treatment for their NP. In accordance with the guidelines approved by the local ethical committee and the Declaration of Human Rights, Helsinki, written informed consent was obtained from all participants.

### 2.3. Evaluation Methods

#### 2.3.1. Demographic Data

All patients were verbally inquired regarding their age, body mass index, and information about when the symptoms onset was. All these data were recorded.

#### 2.3.2. Pain

The severity of pain at rest and during activity was assessed by visual analog scale (VAS). Participants were questioned about their average pain over the last 4 weeks. They were asked to mark the severity of their pain on a 10 cm long line, where 0 represented no pain and 10 stood for vicious pain [8]. The results were recorded in cm.

#### 2.3.3. Neck Disability Index (NDI)

This scale was used to evaluate how the participants' daily life was influenced by their NP. Total score of the scale ranges from 0 to 35, and higher scores indicate higher levels of disability [9].

#### 2.3.4. Tampa Scale of Kinesiophobia (TSK)

This scale was used to assess the patients' fear of pain or reinjury due to movement. It consists of 17 items and assesses various factors of fear/avoidance and injury/reinjury in several activities. Total score of the scale varies between 17 and 68, and higher scores represent higher levels of kinesiophobia [10].

#### 2.3.5. Range of Motion

A universal goniometer was used to assess the ROM of the cervical vertebrae. Cervical flexion, extension, right and left lateral flexion, and right and left rotation movements were measured 3 times in an active manner while the patients were in a comfortable sitting position. The average value of the measurements was recorded as ROM [11]. The pain-free maximum degree of movement for each range was measured in degrees. This method has demonstrated good reliability [12].

#### 2.3.6. Beck Depression Inventory (BDI)

Participants' level of depression was assessed using BDI that consists of 21 categories with 4 options in each category. Each item has a score between 0 and 3, and total score varies from 0 to 63. Score ranges are interpreted as 0–9 points = minor depression, 10–16 points = mild depression, 17–29 points = moderate depression, and 30–63 points = severe depression [13].

#### 2.3.7. Short Form-36 (SF-36)

This form was used to assess the QoL of the participants. This questionnaire consisted of 36 questions that are categorized into 8 groups as follows: physical role functioning, emotional role functioning, bodily pain, energy, social role functioning, mental health, and general health perception. Each category is scored on a 0–100 range, and higher scores indicate better QoL [14].

### 2.4. Treatment Programs

Forty-two older adults who agreed to participate in the study were divided into two groups using a matched randomization method. All participants in both the TP group and the TPMM group were included in a treatment program for 10 sessions.

#### 2.4.1. Traditional Physiotherapy Group

In this study, traditional physiotherapy includes heat modalities, electrotherapy (transcutaneous electrical nerve stimulation (TENS) and ultrasound therapy), and exercises. Patients were asked to lie down in prone, and a pillow was placed under their abdomen for relaxation. We used the hot pack to induce vasodilitation and reduce muscle spasm in this study. A hot pack wrapped in 4 layers of towel was used for 15 minutes to treat for relaxing muscle spasms and for improving soft tissue elasticity [15]. TENS is a simple noninvasive modality and commonly used in both acute and chronic neck pains. The mechanism of analgesia with TENS is described as the “gate control theory” of pain, which is characterized by the modulation of nociceptive input in the dorsal horn of the spinal cord, by peripheral electrical stimulation of large sensory afferent nerves. Alternatively, electrical stimulation of certain receptor sites in the dorsal horn of the spinal cord may release endorphins and produce analgesia that can be reversed by the naloxone. A 50 Hz conventional TENS with a pulse duration <150 microseconds was used in our study. TENS was applied to the painful area of the neck for 20 minutes [16]. Ultrasound therapy, which is used to heat deep tissues, is one of the most important physical treatment methods. Ultrasound increases local metabolism, circulation, regeneration, and extensibility of connective tissue with its assuming thermal and mechanical effects. Ultrasound device (Chattanooga, USA) was used in the study. Ultrasound's gel was applied circularly with a thickness of 2-3 mm. Then, ultrasound with a 4 cm^2^ probe was applied with 1 MHz frequency and 1.5 Wt/cm^2^, for 5 min [17]. Furthermore, massage and exercise were suggested to participants. Classic regional massage was performed on the cervical and thoracic regions. Participants were informed and educated about effective ways of performing their daily life activities. In the context of therapeutic exercises, the older adults were trained for ROM exercises (anterior, lateral, and rotational) and posture exercises (shoulder circumduction, scapular adduction, and pectoral stretching). These exercises were repeated 5 times within the treatment program and 10 times after the program.

#### 2.4.2. Traditional Physiotherapy-Mulligan Mobilization Group

In this group, the MMT was applied in addition to the treatment program applied to the TP group. For two weeks, participants received SNAGs five days per week. According to the MMT, any minor positional fault at a joint can cause a limitation in its physiological movement. The first intervention of the MMT was the application of the natural apophyseal glides (NAGs) applied between C2 and C7. Patients were asked to sit and rest their back against a chair. The mobilization was reapplied by the oscillatory movements and was less than 6 repeats. SNAGs were a combination of mobilization and active movements for the vertebral column. Load-bearing positions were selected and performed at each spinal level. The technique was done without pain at the end of the joint movement [5]. With patients in a seated position, cervical SNAGs were applied with one thumb supported by the other that was placed—depending on the indication—on either the articular pillar or the spinous process of the upper vertebra of the functional spine unit. The therapist applied a passive intervertebral movement which was in a superoanterior direction along the facet plane. The therapist maintained this “glide” as the patient actively moved in any range of physiological movement and then sustained it at the end-range position for a few seconds. The release of the “glide” was when the patient returned to the starting position of the active movement [5]. For two weeks, this mobilization was repeated 6 times per session by a physiotherapist (OB) who holds a certificate in the MMT with 8 years of experience.

### 2.5. Sample Size

In accordance with the study by Ganesh et al., sample size was based on NDI scores in the patient with NP [4]. Their study was designed to investigate the effects of MMT on NP. Large effect size was calculated for this study. Therefore, with a statistically significant level of 5% (*p*=0.05), a statistical power of 80%, an effect size of 0.8, and a minimum of 21 participants were required per group. Allowing for a 10% dropout rate, 47 subjects were recruited into the study.

### 2.6. Statistical Analysis

Statistical analyzes of the study were conducted using the “Statistical Package for Social Sciences” (SPSS) Version 18.0 (SPSS Inc. Chicago, IL, USA). Normal distribution of the data was examined using the “Shapiro–Wilk test.” All outcome analyses were conducted according to the intention-to-treat principle. The “Wilcoxon paired two sample test” was used to compare pretreatment and posttreatment intragroup differences in the findings obtained as a result of the evaluations. “Mann–Whitney *U* Test” was used to compare differences between the two groups.

## 3. Results

**Figure 1:**
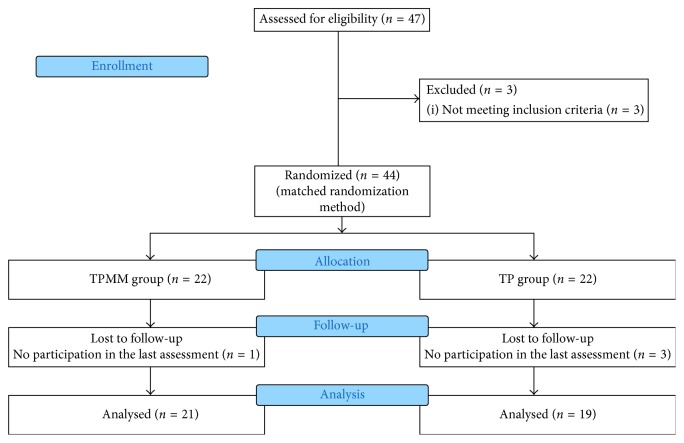
Flow chart of the study.

Among 47 older adults assessed at baseline, 3 were not meeting the inclusion criteria and 4 were lost to follow-up. Finally, the study was completed with 21 older adults in group TPMM, and 19 individuals in group TP (Figure 1). Sociodemographic data of the older adults in both the TP and TPMM groups were similar (*p* > 0.05) (Table 1).

Comparing pretreatment and posttreatment findings indicated that the participants in both groups had a significant decrease in their pain, NDI, BDI, and TSK. They also had significant increase in their ROM and SF-36, except for the physical health condition category for the TPMM group (*p* < 0.05) (Table 2).

Comparing the gains of the participants in the two groups indicated that pain, NDI, right/left neck rotation, left lateral flexion ROM, and mental health subcategory of SF-36 had similar improvement rate in both groups (*p* > 0.05) (Table 3).

However, the two groups were different in terms of ROM (except for right/left neck rotation and left lateral flexion), TSK, BDI, and SF-36 (except for mental health subcategory), in all of which the TPMM group had greater improvements (Table 3).

## 4. Discussion

The results of this randomized, controlled, and double-blinded study showed that all participants had less pain, depression, and kinesiophobia; greater ROM; and better QoL and functional level. It was also found that there was greater improvement in joint ROM (except for right/left rotation and left lateral flexion), kinesiophobia, depression, and QoL (excluding mental health) in the TPMM group compared to the other group.

As in all age groups, NP is a common health problem in the older adults [18]. As a result of the treatment programs of the present study with older adults, NP decreased in a similar way in both groups. In their study on individuals with chronic mechanical NP, Said et al. reported that the MMT had a greater impact on pain reduction compared to the traditional treatment [19]. According to the main explanation provided for the pain-reducing effect of the mobilization, mobilization movements correct positional faults in the bony structure and hence reduce pain [19, 20]. Some studies have reported that spinal manipulative therapy produces a specific hypoalgesic effect. Manipulation-induced hypoalgesia may seem to be nonopioid in nature; that is, it is not reversed by the naloxone and could not improve tolerance to repeated stimulation. It may occur concurrent to changes in sympathetic and motor systems. Furthermore, preliminary evidence indicates that mechanical hypoalgesia is more effective against thermal hypoalgesia in study populations. This specific effect is produced by manipulative therapy [21, 22]. It was believed that the precise mechanism of the sudden development brought about by SNAGs was complex containing many systems including sympathoexcitation and nonopioid hypoalgesia. [23]. El-Sayed et al. was emphasized that the rationale for the technique was initially based on a biomechanical explanation where repositioning of the superior articular facet using a SNAG would cause correction of positional fault, thus resulting in reduced pain and increased ROM in the neck [23]. In accordance with abovementioned studies, results of this study indicated a reduced level of pain in both groups. However, the fact that our participants consisted of older adults suffering NP was an outstanding point of the present study.

One of the most common symptoms of cervical spine problems are restricted ROM [24]. According to the treatment results, there was an increase in the ROM in both groups. However, this increase was found to be greater in the TPMM group except for neck rotation and left lateral flexion. In their randomized controlled study, Gautam et al. divided 30 individuals with NP into 3 groups. They applied the MMT, Maitland technique, and TP to the first, second, and third groups, respectively. They reported that out of the three, the MMT had a greater impact on pain, ROM, and disability [11]. According to Edmonston and Singer, SNAGs are particularly important in painful movement dysfunctions as a result of degenerative changes, as these techniques make pain-free movements possible throughout the available ROM. Furthermore, the potential problems that may occur during passive movements are less likely as the patient is in control of the movement [25]. It is stated that in the MMT, zygapophyseal joints guide the spine, and thus applying NAGs and SNAGs lead to an increase in ROM [26]. The reason for the technique was based on a biomechanical explanation that repositioning the superior articular facet using SNAGs at the beginning would lead to correction of the positional impairment and thus result in pain reduction and increased ROM. Furthermore, normal movement on the articular surface is necessary to maintain the mobility of adjacent nerves that altered biomechanics may affect the nervous outgrowth. Because of this, restoration of normal mechanics in joint space may normalize negative neuron-names that appear as a consequence of limited joint movement [23]. Many studies have indicated a decrease in cervical joint mobility as a result of aging, as well [27–29]. Older adults with neck pain were included in our study, and the MMT were found to improve ROM of the joints. In the literature, however, the MMT seems to be applied to young adults [11, 30]. For this reason, there is a need for studies that investigate the efficacy of the MMT on older adults with NP.

Ganesh et al. divided individuals aged 21–45 years with mechanical neck pain into 3 groups in their studies. They applied Mulligan mobilization to group 1, Maitland mobilization to group 2, and exercise therapy only to group 3. At the end of their studies, they found that manual therapy techniques were not as good as pain relief, increase to ROM and neck disability as compared to exercise (level of evidence = 1C) [4]. Shin and Lee designed a single blind and randomized controlled trial in their study and divided randomly forty patients with headache into the SNAGs group and the control group. Shin and Lee were reported that the SNAGs technique can help to relieve headache and cervical pain in middle-aged women suffering from cervical headache (level of evidence = 1B) [31]. El-Sayed et al. divided randomly patients with radiculopathy whose ages were 40–55 years into the SNAGs + conventional physical therapy group and the conventional physical therapy group in their study. They explained that the SNAGs technique combined with TP is more effective in the rehabilitation program (level of evidence = 1B) [23]. Copurgensli et al. designed a single blind and randomized controlled trial. They were randomly placed into three groups: group 1: conventional rehabilitation; group 2: conventional rehabilitation and MMT; and group 3: conventional rehabilitation and kinesio taping. Results of their study showed that the MMT and kinesio taping have no additional effects on neck pain, muscle strength, and neck-related disability. Furthermore, they said that the use of the MMT and kinesio taping in addition to conventional rehabilitation, the gain in cervical ROM, and deep cervical flexor muscle strength may be increased in patients with cervical spondylosis (level of evidence = 1B) [32]. These studies are generally randomized controlled (level of evidence = 1B) studies in the literature. In comparison studies of the MMT with other treatments there are different opinions about whether it is effective or not [4, 23, 31, 32]. In our study, older adults aged 65 years and over were included, and the MMT has been found to have significant effects on pain, ROM, functional level, kinesiophobia, depression, and QoL. Moreover, the studies in young adults in the literature have been designed nonblindness or single blindness. This study was designed as a double-blinded-randomized controlled trial. To the best of our knowledge, there were few studies which compare the effects of the MMT on pain, ROM, functional level, kinesiophobia, depression, and QoL in older adults with chronic neck pain.

It has been shown that there is an important relationship between pain and kinesophobia in individuals suffering NP. As a result of our study, it was determined that fear of movement decreased in both groups. This decrease was more pronounced in the TPMM group. It is also stated that, in case of NP, ROM in the cervical region decreases, movements are slower than normal, and proprioception is impaired [24, 30]. It is thought that any increase in ROM results in an increase in the proprioceptive sensation in the neck region, which may result in reduced kinesophobia in patients.

NDI was used in the present study to assess the patients' disabilities in daily life due to their NP. According to our posttreatment evaluations, NDI results had improved in both TP and TPMM groups in a similar manner. Our results are in agreement with Sudarshan, who applied a simultaneous combination of neurodynamic mobilization and SNAGs and reported immediate improvement in VAS, cervical ROM, and NDI. In our study, similar development was achieved in both groups [33]. This is thought to be due to the fact that older adults are able to perform their daily life activities better as a result of reduced pain.

SF-36 was used to assess the QoL of the participants. This questionnaire was developed specifically to assess QoL in patients with physical illnesses [14]. At the end of our treatment programs, there was an increase in QoL in both the TP and TPMM groups, which was found to be higher in the TPMM group except for the mental health score. Maiers et al. investigated the effects of spinal manual therapy (cervical joint and soft tissue mobilization) and exercises in the older adults with chronic NP. They reported minor improvement in QoL following the treatment; however, this improvement was not statistically significant [18]. Even though the present study is similar to the one by Maiers et al. in terms of patient population, we achieved greater gains in QoL of our participants. The TPMM group showed more pronounced improvement in QoL (except for the mental health scores), and this is thought to be a result of higher ROM and reduced pain, both of which have positive effects on QoL. These two parameters are more significantly gained in the TPMM group.

BDI was used to determine the risk of depression in patients and/or to measure the level of depressive symptoms and the change in its severity [13]. As a result of this study, both the TP and TPMM groups showed a decrease in depression levels, and this decrease was found to be higher in the TPMM group.

The most important outcome of the present study is that the MMT can be safely applied in older adults with NP without harming the patients. In addition, functional limitations in older adults with NP were reduced, and pain-free ROM was obtained. Although there are some studies in the literature evaluating the efficacy of the MMT in individuals suffering NP, there are no studies investigating the efficacy of this technique on older adults. Two strengths of this study are that the patient group consists of older adults and that it is a random-controlled double-blind study.

Long-term effects of the MMT in older adults with NP are not investigated, which is a limitation of the present study. To have more precise results, it is necessary to continue long-term follow-up evaluations of the patients to investigate the rate of recurrence in each group.

## 5. Conclusion

According to the findings of this study, applying the MMT in older adults with NP has positive effects on pain, ROM, functional level, kinesiophobia, fear of movement, ES, and QoL.

## Figures and Tables

**Table 1:** Demographic information of individuals.

	TP group median (IQR)	TPMM group median (IQR)	*p*
Age (years)	67 (65.5–72)	69 (65–70.5)	0.575
BMI (kg/m^2^)	27.78 (24.675–28.545)	28.34 (24.245–30.01)	0.763
Duration of diagnosis (years)	10 (6–12)	9 (7–12)	0.453

IQR: inter quartile range; BMI: body mass index.

**Table 2:** Comparing pretreatment and posttreatment participants in both groups.

		TP group			TPMM group			*p*2
		Before median (IQR)	After median (IQR)	*p*1	Before median (IQR)	After median (IQR)	*p*1	
VAS (0–10)								
Rest		5 (4–7)	2 (0–3)	0.007^*∗*^	4 (2–5.5)	0	0.002^*∗*^	0.171
Activity		7 (4–8.5)	2 (0–4)	0.005^*∗*^	7 (5–8)	1 (0–2)	0.002^*∗*^	0.224
ROM								
Cervical flexion		34 (32.2–36.3)	41 (39.2–43.3)	0.005^*∗*^	35 (33.3–36.5)	46 (40.8–47.5)	0.003^*∗*^	0.165
Cervical extension		35 (34.6–36.2)	40 (35.4–42.3)	0.005^*∗*^	33 (32.5–36.4)	41 (37.4–45.2)	0.003^*∗*^	0.089
Cervical lateral flexion	Right	32 (30.2–33.4)	38 (35.7–39.7)	0.005^*∗*^	33 (30.4–38.5)	42 (40.2–48.5)	0.002^*∗*^	0.153
	Left	32 (29.6–34.3)	37 (34.5–39.6)	0.005^*∗*^	34 (31.6–36.3)	40 (38.4–45.7)	0.003^*∗*^	0.083
Cervical rotation	Right	42 (39.2–43.1)	45 (40.01–44.8)	0.007^*∗*^	45 (39.6–46.5)	52 (45.7–53.5)	0.012^*∗*^	0.091
	Left	39 (34.4–42.5)	42 (39.2–44.03)	0.008^*∗*^	35 (32.7–36.5)	48 (45.5–52.4)	0.003^*∗*^	0.079
NDI (0–35 points)		17 (15–18)	7 (4–8)	0.005^*∗*^	18 (16–20)	5 (4–6)	0.002^*∗*^	0.116
TSK (17–68 points)		41 (40–41)	38 (37–41)	0.005^*∗*^	40 (39–42)	36 (35–40)	0.003^*∗*^	0.057
BDI		15 (7–19)	7 (3–9)	0.005^*∗*^	13 (10–14)	6 (4–8)	0.002^*∗*^	0.098
Quality of life (SF-36)	Physical component	35.8 (33–41.2)	40.4 (40.5–42.7)	0.005^*∗*^	36.4 (34.6–36.9)	42.3 (41.8–46.5)	0.182	0.091
	Mental component	39.8 (37.5–43.6)	43.3 (40.6–46.3)	0.005^*∗*^	38.7 (36.5–40.2)	45.7 (41.5–48.7)	0.003^*∗*^	0.131
	Total	70.5 (69.2–76.7)	80.3 (78–85.5)	0.005^*∗*^	72.4 (70.2–75.9)	88.2 (85.4–89.1)	0.002^*∗*^	0.052

TP: traditional physiotherapy, TPMM: traditional phyisotherapy + Mulligan mobilization, IQR: interquartile range, VAS: visual analog scale, ROM: range of motion, NDI: neck disability index, TSK: Tampa scale of kinesiophobia, BDI: Beck depression inventory, SF-36: Short Form-36. *p*1 denotes the differences between before and after treatment scores for both groups with using “Wilcoxon paired two sample test,” and *p*2 denotes the differences between the baseline scores of two groups with using “Mann–Whitney *U* test.” ^*∗*^*p* < 0.05.

**Table 3:** Comparing the gains of the participants in both groups.

		TP group Δ median (IQR)	TPMM group Δ median (IQR)	*p*
VAS (0–10)				
Rest		−3 (−6 to −3)	−4 (−6 to −2)	0.862
Activity		−5 (−5 to −4)	−6 (−6 to −3)	0.083
ROM				
Cervical flexion		6.4 (4.2–6.9)	10.2 (8.3–12.4)	≤0.001^*∗*^
Cervical extension		5.3 (3.7–6.4)	8.4 (5.8–9.7)	≤0.001^*∗*^
Cervical lateral flexion	Right	6 (4.4–7.1)	9 (8.01–11.2)	0.004^*∗*^
	Left	5 (3.5–6.8)	6 (5.4–8.2)	0.089
Cervical rotation	Right	3 (2.7–4.7)	7 (5.6–8.3)	0.527
	Left	3 (2.9–4.5)	13 (10.5–15.6)	0.354
NDI (0–35 point)		−10 (−12 to −8)	−13 (−14 to −7)	0.335
TSK (17–68 point)		3 (4–6)	5 (4–8)	0.006^*∗*^
BDI		−8 (−11 to −4)	−7 (−10 to −4)	0.007^*∗*^
Quality of life (SF-36)	Physical component	4.5 (2.1–6.2)	5.9 (4.3–6.7)	0.002^*∗*^
	Mental component	4.7 (3.2–10.43)	7.3 (5.25–9.82)	0.092
	Total	10.5 (4.3–12.4)	16.1 (8.9–20.21)	0.002^*∗*^

TP: traditional physiotherapy, TPMM: traditional phyisotherapy + Mulligan mobilization, VAS: visual analog scale, ROM: range of motion, NDI: neck disability index, TSK: Tampa scale of kinesiophobia, BDI: Beck depression inventory, SF-36: Short Form-36. ^*∗*^*p* < 0.05.
